# Remdesivir Is Effective in Combating COVID-19 because It Is a Better Substrate than ATP for the Viral RNA-Dependent RNA Polymerase

**DOI:** 10.1016/j.isci.2020.101849

**Published:** 2020-11-28

**Authors:** Tyler L. Dangerfield, Nathan Z. Huang, Kenneth A. Johnson

**Affiliations:** 1 Department of Molecular Biosciences, The University of Texas at Austin, 100 W. 24th Street, Stop 5000, MBB 3.122, Austin, TX 78712, USA

**Keywords:** Biological Sciences, Biochemistry, Molecular Biology, Molecular Biology Experimental Approach

## Abstract

COVID-19 is caused by the severe acute respiratory syndrome coronavirus 2 (SARS-CoV-2) and is currently being treated using Remdesivir, a nucleoside analog that inhibits the RNA-dependent-RNA polymerase (RdRp). However, the enzymatic mechanism and efficiency of Remdesivir have not been determined, and reliable screens for new inhibitors are urgently needed. Here we present our work to optimize expression in *E. coli*, followed by purification and kinetic analysis of an untagged NSP12/7/8 RdRp complex. Pre-steady-state kinetic analysis shows that our reconstituted RdRp catalyzes fast (*k*_*cat*_ = 240–680 s^−1^) and processive (*k*_*off*_ = 0.013 s^−1^) RNA polymerization. The specificity constant (*k*_*cat*_*/K*_*m*_) for Remdesivir triphosphate (RTP) incorporation (1.29 μM^−1^s^−1^) is higher than that for the competing ATP (0.74 μM^−1^ s^−1^). This work provides the first robust analysis of RNA polymerization and RTP incorporation by the SARS-CoV-2 RdRp and sets the standard for development of informative enzyme assays to screen for new inhibitors.

## Highlights

• Co-expression of NSP12/7/8 with chaperones in *E. coli* gives soluble SARS CoV2 RdRp
• Tag-free RdRp complex catalyzes fast and processive RNA polymerization
• Polymerization rates are sufficient to replicate the 30 kb genome in 2 min
• Remdesivir is incorporated with a specificity constant twice that observed for ATP

## Graphical Abstract

**Figure:**
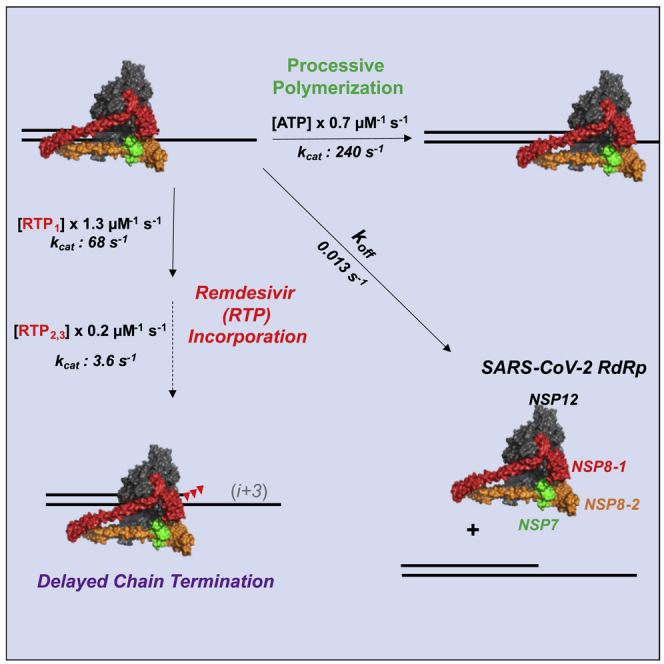

## Introduction

A small outbreak of SARS-CoV-2 originated in Wuhan, China in December 2019 (Zhu et al., 2020) and swiftly led to a devastating global pandemic due to the highly contagious and virulent nature of this novel virus. Besides the push to develop a safe and widely available vaccine, work proceeds to develop direct-acting antiviral drugs. The viral RNA-dependent RNA polymerase (RdRp), consisting of non-structural proteins (NSP) 7, 8, and 12, is an important drug target as it catalyzes replication of the viral genome. Viral RNA and DNA polymerases are proven effective targets for the treatment of viral infections (Deval et al., 2014). For example, inhibitors of HIV reverse transcriptase are the cornerstone of multidrug therapies to treat HIV infections (Hurwitz and Schinazi, 2012), and inhibitors of the hepatitis C virus (HCV) RdRp are central to treating HCV infections (Deval et al., 2014). Although there is a wealth of structural data on the SARS CoV-2 RdRp (Ferron et al., 2018; Hillen et al., 2020; Kirchdoerfer and Ward, 2019; Yin et al., 2020), there are no accurate kinetic studies to establish the mechanistic basis for effective inhibition.

The antiviral compound Remdesivir is an ATP analog, originally developed to treat Ebola infections (Pardo et al., 2020). On 22 October 2020 Remdesivir was approved by the FDA for treatment of COVID-19 based on promising clinical results and initial approval for compassionate use (Grein et al., 2020). It seems unlikely that an ATP analog could be effective because of the high concentrations of competing ATP *in vivo* but Remdesivir seems to show promise despite this hurdle. Therefore, accurate data are required to understand the mechanistic and kinetic basis for the effectiveness of Remdesivir and to use that knowledge to screen for and develop additional antiviral compounds. Although drug screening based on propagation of virus in cell culture has been effective in developing new therapies, this method is expensive and time consuming, making it difficult to screen millions of compounds in the search for new inhibitors. Assays based on analysis of well-defined kinetics of polymerization catalyzed by the viral RdRp *in vitro* could provide a critical component to find new drug candidates. However, preliminary studies attempting to address the kinetics and mechanism of Remdesivir incorporation were flawed because they were conducted using substandard enzyme preparations requiring 30 min of incubation to see a product band on a gel (Gordon et al., 2020b). Moreover, in lieu of accurate rate measurements, a surrogate assay was developed based on fractional extension of a short primer after it was labeled by incorporation of one nucleotide and then subsequently extended by incorporation of Remdesivir or ATP (Gordon et al., 2020b). Expression and purification of a viral polymerase for use in kinetic analysis must meet standards of purity and activity for the results to be valid. In particular, a polymerase must be capable of replicating the RNA at rates sufficient to account for the time required for viral replication *in vivo,* be fully active, and be free of contaminating nuclease activity. Most enzyme purification strategies rely on the use of tagged enzyme without regard to the effects of the tags on activity. For example, in prior work on HIV reverse transcriptase, purification without tags was essential for getting the most active enzyme preparations (Lu et al., 2010). Prior efforts to study the coronavirus RdRp *in vitro* have mostly relied on baculovirus-infected insect cell expression (Gordon et al., 2020b; Hillen et al., 2020; Yin et al., 2020) to overcome the low solubility of NSP12 when overexpressed in *E. coli*. However, baculovirus expression of protein is expensive, time consuming, and the low yield usually requires the use of tagged protein.

Prior coronavirus RdRp inhibition studies have relied on steady-state kinetic methods (Gordon et al., 2020b). However, steady-state kinetic assays do not yield meaningful results when applied to processive enzymes due to the slow, rate-limiting dissociation of the enzyme from the primer/template after the much faster rate of nucleotide incorporation (Kuchta et al., 1987). Rate-limiting dissociation masks inherent properties of nucleoside analogs, thereby misdirecting drug design studies. To circumvent problems with the steady-state approach and provide a robust system for studies on the RdRp complex, here we present pre-steady-state experiments to accurately monitor the kinetics of polymerization and quantify the kinetics of Remdesivir incorporation relative to ATP. This work is based on our optimization of the expression and purification of tag-free SARS-CoV-2 RdRp complex in *E. coli*, yielding protein that is capable of fast processive synthesis, as revealed by the pre-steady-state kinetic measurements. We reveal key features of Remdesivir and lay the foundation for robust assays to screen for and optimize new inhibitors for treating SARS-CoV-2 and future novel coronavirus infections.

## Results

### Co-Expression of SARS-CoV-2 RdRp Subunits with Chaperones Gives Soluble Expression in *E. coli*

The SARS-CoV-2 RdRp complex, responsible for the replication of the positive-sense single-stranded RNA genome, consists of the NSP12 catalytic subunit with accessory proteins NSP7 and NSP8 (Hillen et al., 2020). Most mechanistic studies have been performed using protein purified from baculovirus-infected insect cells with histidine or other tags used in purification (Gordon et al., 2020b; Hillen et al., 2020; Yin et al., 2020). Because of the many advantages of *E. coli* expression, we set out to optimize expression of the RdRp complex in *E. coli*. Initial expression of untagged NSP12 alone yielded virtually all of the protein in inclusion bodies at all temperatures tested from 8–37°C (data not shown). A recent paper (Shannon et al., 2020) expressed a histidine-tagged NSP12 in *E. coli* by co-expression of chaperones from a separate plasmid, pG-Tf2 (Nishihara et al., 2000). This plasmid contains tetracycline inducible copies of Tf, GroEL, and GroES chaperones. When we tested expression of NSP12 alone with pG-Tf2, we observed a slight increase in solubility; however, the majority of the protein was still insoluble (data not shown).

**Figure 1:**
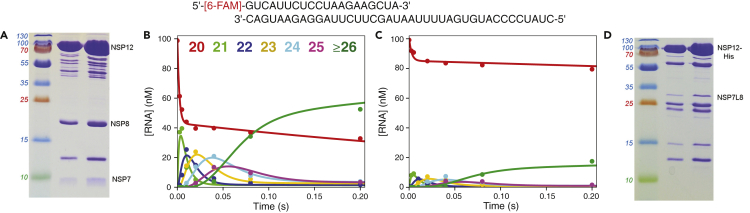
Tag-Free SARS-CoV-2 NSP12/7/8 Complex Has Higher Activity than the His-tagged NSP12/NSP7L8 Complex Top: RNA substrate. The sequence was copied from the SARS-CoV-2 genome preceding the poly(A) tail. The primer strand was synthesized with a 5’-[6-FAM] label to monitor reaction kinetics by capillary electrophoresis. (A) Gel showing purified NSP12/7/8 complex. Molecular weight markers are given to the left of the gel and 2 dilutions of the purified product are shown. Bands corresponding to NSP7, NSP8, and NSP12 are labeled to the right of the gel. (B) NSP12/7/8 complex activity assay. A solution of 2 μM NSP12/7/8 complex, 5 μM NSP8, 100 nM FAM-20/40 RNA, and 5 mM Mg^2+^ was mixed with 250 μM each of ATP, CTP, and UTP to start the reaction. Approximately 60% of the starting RNA was turned over to product in the first turnover. Colors for each RNA length are given at the top of the panel. (C) NSP12-His/NSP7L8 complex activity assay. A solution of 2 μM NSP12-His/NSP7L8 complex, 5 μM NSP8, 100 nM FAM-20/40 RNA, and 5 mM Mg^2+^ was mixed with 250 μM each of ATP, CTP, and UTP to start the reaction. Around 15% of the RNA was converted to product during a single turnover. RNA products of various lengths are colored as in (A). To a first approximation, note that the midpoint in forming the final product (12 nt incorporated) is ~0.06 s, giving an average polymerization rate of ~200 s^-1^. (D) Gel showing purified NSP12-His/NSP7L8 complex. Molecular weight markers are given to the left of the gel and 2 dilutions of the purified product are shown. Bands corresponding to NSP12-His and NSP7L8 are shown to the right of the gel.

Co-expression of proteins with their cellular protein partners has been an effective strategy to improve solubility in some difficult recombinant protein expression systems (Chiu et al., 2006). To test this strategy for the SARS-CoV-2 replication complex, an expression plasmid pQE-(NSP12)-pcI^ts,ind+^-(NSP7-NSP8) was constructed so that it contains codon-optimized genes for NSP12, NSP7, and NSP8 in a single expression plasmid (Figure S1A). NSP12 was cloned under the IPTG inducible T5 promoter/lac operator from the pQE-30 parent plasmid, and NSP7 and NSP8 were cloned as a bicistronic operon under the temperature/nalidixic acid inducible bacteriophage λ promoter from the pcI^ts,ind+^ backbone (Brandis and Johnson, 2009). This plasmid also has the kanamycin resistance gene, the high copy number pUC origin of replication, and on-board λcI^ts,ind+^ and lacI repressors so protein can be expressed in any recA^+^ *E. coli* strain (Brandis and Johnson, 2009). For comparison of the untagged NSP12/7/8 complex to a histidine-tagged NSP12 and modified NSP7/NSP8 construct containing a GSGSGS linker used in a previous study (Shannon et al., 2020), the plasmid pQE-(NSP12-TEV-8xHis)-pcI^ts,ind+^-(NSP7L8) was created (Figure S2). We prepared BL21 *E. coli*/pG-Tf2 harboring either pQE-(NSP12)-pcI^ts,ind+^-(NSP7-NSP8) or pQE-(NSP12-TEV-8xHis)-pcI^ts,ind+^-(NSP7L8) to test solubility of the complexes when co-expressed with chaperones from pG-Tf2. The chaperone/co-expression strategy yielded virtually all soluble protein for the untagged construct (Figures S3A and S3B) and mostly soluble protein for the his-tagged NSP12/NSP7L8 complex (not shown). We purified the his-tagged complex on a nickel column and the untagged complex with a series of five different columns as described in the Transparent Methods section. The final step in the purification of the untagged complex was a size exclusion column, which separated NSP12 alone and complexes of NSP12/NSP7 and NSP12/NSP8 from the NSP12/NSP7/NSP8 complex. It is difficult to tell the exact stoichiometry from bands on a gel; however, the presence of smaller complexes lacking one or more subunits during size exclusion chromatography suggests the pooled complex of NSP12/NSP7/NSP8 has each subunit at least at a 1:1:1 ratio. For the his-tagged NSP12, the NSP7L8 polypeptide co-purified on the nickel column (Figure 1D) and for the untagged NSP12, NSP7, and NSP8 were all visible in the final purified product (Figure 1A). Becasue NSP8 has been shown to bind with two molecules per one molecule of NSP7 and NSP12 (Hillen et al., 2020), NSP8 was added in excess to the purified replication complex to saturate NSP8 binding. NSP8 was cloned into the pcI^ts,ind+^ backbone (Figure S1B) and was overexpressed in the soluble fraction with heat induction in *E. coli* and purified with four different columns (Figure S4) as described in the Transparent Methods section.

### Tag-free SARS-CoV-2 NSP12/7/8 Complex Has Higher Activity Than His-Tagged NSP12/NSP7L8 Complex

We initially tested the primer extension activity of the various purified SARS-CoV-2 proteins with a pre-steady-state rapid quench experiment. The RNA substrate used (Figure 1) in this paper consists of a 20 nt 5’-[6-FAM] labeled primer, annealed to a 40 nt template with the sequence from the SARS-CoV-2 genome (preceding the poly(A) tail) (Ferron et al., 2018; Ma et al., 2015). The experiment was performed with a large excess of enzyme over RNA. NSP8 was added to 5 μM for initial experiments, which is approximately 10 times the reported *K*_*d*_ for NSP8 binding to NSP12 (Peng et al., 2020). We tested both the untagged NSP12/7/8 and histidine-tagged NSP12-His/NSP7L8 protein preparations for primer extension activity. Reaction products were diluted into formamide and separated by capillary electrophoresis at 65°C in nanoPOP-6 polymer, which was sufficient to completely denature the RNA for separation (Figure S5).

For the untagged protein, a solution containing 2 μM NSP12/7/8 complex, 5 μM NSP8, 100 nM FAM-20/40 RNA, and 5 mM Mg^2+^ was allowed to equilibrate for 10–60 min (the time to complete the experiment), then was mixed with 250 μM ATP, CTP, and UTP to start the reaction. Adding three of the four nucleotides afforded extension of the primer by 12 residues before a C in the template was encountered, terminating synthesis. Following the addition of the three nucleotides, rapid extension of the RNA primer to form the 32 nt product was observed in 0.2 s (Figure 1B). Under the parallel conditions with the NSP12His/NSP7L8 complex, we also observed rapid formation of the 32 nt product, but only ~15% of the RNA primer was extended in a single turnover compared with 60% with the untagged NSP12/7/8 complex (Figure 1C), suggesting either a lower fraction of active protein, weaker RNA binding by the tagged polymerase and/or suboptimal concentration of the NSP7L8 accessory protein. No degradation of the primer by RNases was detected for either tagged or untagged RdRp complex during the roughly one-hour preincubation of the enzyme with RNA in the presence of Mg^2+^ while performing the experiment, indicating there was negligible RNase contamination in either enzyme preparation. It was previously reported for the SARS-CoV NSP12His/NSP7L8 complex that the activity was higher than the NSP12/7/8 complex (Subissi et al., 2014), presumably because the linked NSP7L8 overcame the weak binding of NSP7 and NSP8 to NSP12. However, that was not the case in our hands. The lower activity could be attributed to a number of factors including the histidine tag, the number of columns used for purification, lack of supplemented NSP7L8, etc. We have not repeated the purification and characterization of the tagged enzyme and we did not pursue further optimization of the histidine-tagged complex. Therefore, we cannot definitively conclude that the tag or the NSP7L8 variant interfered with activity; rather, our results serve as a cautionary tale. Because the untagged protein had higher activity and more closely mimics the natural protein, we opted to use the untagged RdRp complex for the remainder of our experiments.

As a control experiment, we also tested NSP8 alone for primer extension activity as a paper on the SARS-CoV (te Velthuis et al., 2012) suggested NSP8 has primer extension activity. A solution of 5 μM NSP8, 100 nM FAM-20/40 RNA, and 5 mM Mg^2+^ was mixed with 250 μM each of ATP, CTP, and UTP to start the reaction. Reactions were quenched with EDTA and time points were analyzed by capillary electrophoresis. No primer extension was observed on the timescale of the experiment, and no degradation of the primer strand occurred during the preincubation of the enzyme with the RNA (data not shown). This indicates that under our experimental conditions, NSP8 does not have measurable primer extension activity and that there is no detectable RNase activity in our tag-free preparation of NSP8.

### Pre-steady-state Kinetics of UTP Incorporation

**Figure 2:**
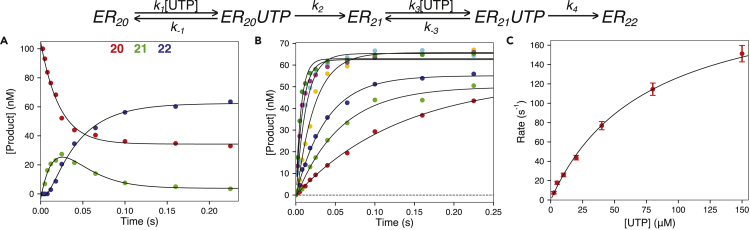
Pre-steady-state Kinetics of UTP Incorporation Scheme: Kinetics of sequential UTP incorporations *ER*_n_ is the enzyme-RNA complex *n* nucleotides in length. *k*_*1*_ and *k*_*3*_ are *k*_*cat*_/*K*_*m*_ for the first and second UTP incorporation, respectively, whereas *k*_*2*_ and *k*_*4*_ define *k*_*cat*_ for the first and second UTP incorporation, respectively. Experiment: a mixture containing 2 μM NSP12/7/8 complex, 6 μM NSP8, 100 nM FAM-20/40 RNA, and 5 mM Mg^2+^ was mixed with varying concentrations of UTP (2.5–150 μM) to start the reaction. (A) UTP incorporation reaction at 20 μM UTP. The fits to double exponential functions are shown by the black lines going through the data points for each RNA species: 20 nt (red), 21 nt (green), and 22 nt (blue). (B) Total product versus time at varying UTP concentrations. The sum of products 21 and 22 nt in length was plotted versus time for reactions performed at various UTP concentrations. The fits to single exponential functions are shown as the black lines going through the data points, with different colors for different concentrations (red to dark green: 2.5, 5, 10, 20, 40, 80, 150 μM). (C) Rate of product formation versus UTP concentration. Rates and standard errors (bars) were derived from the singe exponential fit of the data in (B). This simple analysis illustrates the saturation of the rate versus nucleotide concentration with estimates of *k*_*cat*_ = 220 ± 23 s^−1^ and *K*_*m*_ = 74 ± 11 μM. Better estimates were derived from global data fitting as shown in Figure 3 and summarized in Table 1.

The next experiment was designed to measure the UTP concentration dependence on the rate of polymerization. A pre-equilibrated mixture containing 2 μM NSP12/7/8 complex, 6 μM NSP8, 100 nM FAM-20/40 RNA, and 5 mM Mg^2+^ was mixed with varying UTP concentrations (2.5–150 μM) to start the reaction. Reactions were quenched and analyzed as in Figure 1. Because there are two sequential templating A's, two UTPs were incorporated in rapid succession, as shown in Figure 2A. Data for the total amount of product at each nucleotide concentration are shown in Figure 2B—by summing the 21 and 22 nt products, we define the kinetics of the first UTP incorporation. Figure 2C shows the observed rate versus UTP concentration for the first UTP incorporation, obtained by fitting the data in Figure 2B to a single exponential function. Conventional equation-based fitting of the data in Figure 2C using a hyperbolic function provides estimates for the parameters *k*_*cat*_ = 220 ± 23 s^−1^ and *K*_*m*_ = 74 ± 11 μM for incorporation. To achieve more accurate results, the parameters reported in this paper were obtained by global data fitting based on numerical integration of the rate equations using KinTek Explorer to derive estimates for *k*_*cat*_ and *K*_*m*_ directly (Johnson, 2009, 2019; Johnson et al., 2009b). Data fitting by simulation also allows resolution of multiple sequential polymerization reactions during processive synthesis (Figure 2), and confidence contour analysis affords realistic error estimates based on the extent to which each parameter is constrained by the data (Johnson et al., 2009a). The scheme in Figure 2 shows the model used for fitting data in KinTek Explorer to extract *k*_*cat*_ (*k*_*2*_ and *k*_*4*_), and *K*_*m*_ (((*k*_*-1*_+*k*_*2*_)/*k*_*-1*_), and ((*k*_*-3*_+*k*_*4*_)/*k*_*3*_))) for the two UTP incorporations. The value of *k*_*cat*_/*K*_*m*_ was then calculated from the estimates for *k*_*cat*_ and *K*_*m*_. The parameters *k*_*cat*_/*K*_*m*_, *k*_*cat*_, and *K*_*m*_ for each UTP incorporation derived from the simulation-based data fitting are summarized in Table 1. Of particular importance, note that the specificity constant is defined by *k*_*cat*_*/K*_*m*_.

**Table 1:** Kinetic Parameters for Nucleotide Incorporation^a^

Nucleotide	*k*_*cat/*_*K*_*m*_ (*μM*^*−1*^*s*^*−1*^)	*k*_*cat*_ (*s*^*−1*^)	*K*_*m*_ (*μM*)
UTP-1	2.3 ± 0.2	308 ± 17	130 ± 9
UTP-2	1.7 ± 0.5	680 ± 130	400 ± 80
ATP	0.74 ± 0.16	240 ± 30	320 ± 50
RTP-1	1.29 ± 0.06	68 ± 2	53 ± 2
RTP-2	0.19 ± 0.01	3.62 ± 0.04	19 ± 1
RTP-3	0.18 ± 0.01	3.76 ± 0.07	21 ± 2

a Rate constants were obtained by globally fitting data using KinTek Explorer. Standard errors were obtained from upper and lower limits derived by nonlinear regression analysis backed up by confidence contour analysis (Figures 3 and 5). In the nucleotide column, numbers next to the nucleotide represent each sequential incorporation where multiple incorporations were resolved by the data. Values for *K*_*m*_ and *k*_*cat*_*/K*_*m*_ were calculated from the intrinsic rate constants derived in data fitting as described in the Methods. Values for *k*_*cat*_ were derived directly in fitting as the intrinsic rate constant for incorporation (Johnson, 2019; Kellinger and Johnson, 2010).

### SARS-CoV-2 RdRp Complex Weakly Binds RNA Substrate

Because only 60% of the RNA primer was extended in the previous experiments in spite of a large excess of enzyme over the RNA substrate, the following experiment was performed to differentiate low active enzyme concentration from weak binding to the RNA. A mixture of varying concentrations of the NSP12/7/8 complex (0.2–10 μM), 20 μM NSP8, 200 nM FAM-20/40 RNA, and 5 mM Mg^2+^ was mixed with 150 μM UTP to start the reaction. Reactions were quenched with EDTA, and the amount of total extended primer was determined by capillary electrophoresis. The concentration of product formed versus time at different enzyme concentrations is shown in Figure 3D. The amplitude versus nominal enzyme concentration graph (not shown) fits a hyperbola (*K*_*d*_ = 1.9 ± 0.2 μM), signifying that the binding of the enzyme to the RNA is weak compared with the fixed RNA concentration (200 nM) in the titration. This experiment suggests that the amplitude of 60 nM in Figure 2B is mostly due to weak RNA binding with some effect potentially from inactive enzyme. The *K*_*d*_ estimated from the global fit by simulation was 1.8 ± 0.2 μM for the enzyme binding to RNA, assuming 80% of the enzyme is active, as described below. This information aids in designing additional experiments in this paper, as the amount of product expected to form in a single turnover can be estimated based on the measured *K*_*d*_ for RNA binding.

**Figure 3:**
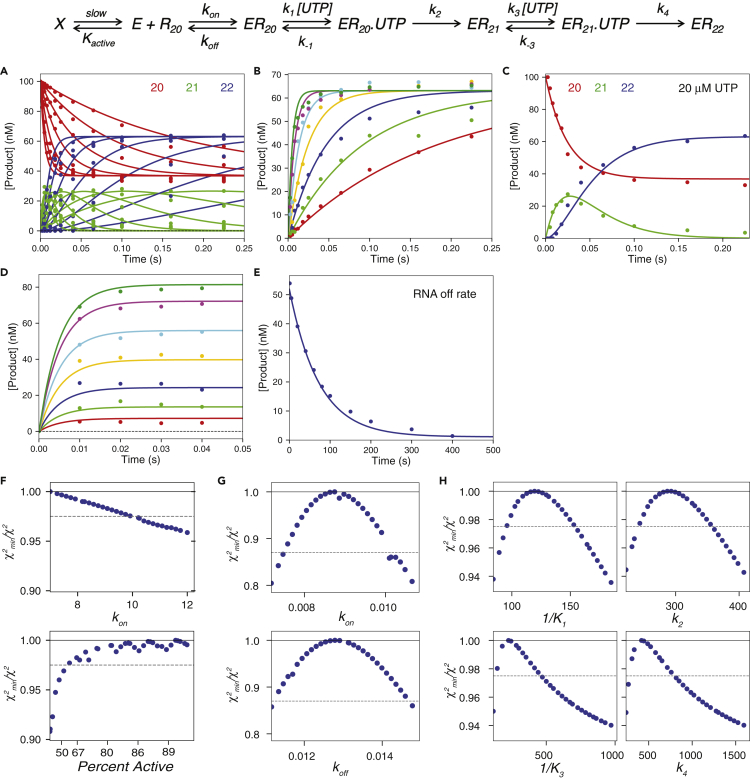
SARS-CoV-2 RdRp Complex Weakly Binds an RNA Substrate and Dissociates at a Very Slow Rate Scheme: *ER*_*n*_ is the enzyme-RNA complex with RNA *n* nucleotides in length *k*_*off*_ is the RNA dissociation rate and *k*_*on*_ is the apparent RNA association rate. The ratio of *k*_*off*_/*k*_*on*_ gives the *K*_*d*_ or the apparent equilibrium binding constant. Kinetic constants for UTP incorporation are labeled as in the scheme in Figure 2. *K*_*active*_ is the equilibrium constant for enzyme going from the active E state to the inactive X state, which occurs during the pre-equilibration step to estimate the active enzyme concentration, as described in the Transparent Methods. Experiments in panels A–C are the same as in Figure 2. (A) UTP incorporation, concentration dependence. Data for the 20, 21, and 22 nt RNA are shown in red, green, and blue, respectively. (B) UTP incorporation, concentration dependence. The concentration of total product versus time is plotted for each UTP concentration. (C) UTP incorporation at 20 μM UTP. RNA of different lengths is colored as in (A). (D) UTP incorporation, enzyme titration. A mixture of 0.2–10 μM NSP12/7/8 complex, 20 μM NSP8, 200 nM FAM-20/40 RNA, and 5 mM Mg^2+^ was mixed with 150 μM UTP to start the reaction. The amount of total product formed was plotted versus time for each enzyme concentration and fit by simulation in KinTek Explorer. (E) RNA dissociation rate experiment. A solution containing 1.25 μM NSP12/7/8 complex, 6 μM NSP8, 100 nM FAM-20/40 RNA, and 5 mM Mg^2+^ was allowed to equilibrate for 30 min, then mixed with 2 mg/mL heparin in the first mixing step using the RQF-3 rapid-quench flow instrument. After the designated first mixing time (shown in the figure), the reaction was mixed with 125 μM UTP (concentration after dilution) from the quench syringe, held in the exit line for 50 milliseconds, then the reaction was quenched by mixing with EDTA to a final concentration of 0.3 M in a collection tube. The time axis in the figure is the time allowed for RNA dissociation after mixing with heparin trap, before the addition of nucleotide. The best fit by simulation is shown as the solid line yielding an RNA dissociation rate of 0.013 ± 0.001 s^-1^ (F) Confidence contours used to estimate fraction active enzyme. Data in panels A–E were fit globally while allowing the equilibrium constant, *K*_*active*_ to vary, along with *k*_*on*_ (which compensates for variable active enzyme concentration). The data were fit to extract the percent of active enzyme and the corresponding *k*_*on*_ values, as described in the Transparent Methods. All other rate constants were also allowed to vary, except *k*_*1*_ and *k*_*3*_ that were locked at 100 μM^−1^s^−1^. Data in the panel show that the enzyme is at least 60% active. (G) Confidence contours to define *k*_*on*_ and *k*_*off*_. Data in panels D and E were fit assuming 80% active enzyme with all other rate constants locked at their best-fit values. This analysis give *k*_*on*_ = 0.008 ± 0.001 μM^−1^s^−1^ and *k*_*off*_ = 0.013 ± 0.001 s^−1^ and *K*_*d*_ for RNA binding of 1.8 ± 0.2 μM. (H) Confidence contours for UTP binding and incorporation. Date in panels A–C were fit assuming 80% active enzyme and with *k*_*on*_ and *k*_*off*_ locked at their best fit values. The values for *k*_*1*_ and *k*_*3*_ were locked at 100 μM^−1^s^−1^. For each contour, the dashed line shows the χ^2^ threshold. The smooth lines in each panel were derived from the global data fit. Rate constants derived from global data fitting, assuming 80% active enzyme, are given in Table 1.

### SARS-CoV-2 RdRp Complex Dissociates Very Slowly from the RNA Substrate

Success of the experiment described in Figure 3D depends on a slow RNA dissociation rate. To measure the rate of RNA dissociation from the NSP12/7/8-RNA complex, a quench flow double-mixing experiment was performed. A solution of 1.25 μM NSP12/7/8 complex, 6 μM NSP8, 100 nM FAM-20/40 RNA, and 5 mM Mg^2+^ was allowed to equilibrate for 30 min, then mixed with 2 mg/mL heparin to start the reaction. The use of 2 mg/mL heparin has been shown to be an effective concentration (Donlin et al., 1991) as a trap for enzyme dissociated from the nucleic acid substrate for other polymerases, and the fact that the amount of product tends toward 0 indicates that heparin is an efficient trapping agent for this enzyme (Figure 3E). This solution was incubated for various amounts of time (mixing step 1) in the reaction loop before mixing with 150 μM UTP and 2 mg/mL heparin from the quench syringe and then allowed to react for 50 ms (mixing step 2). The reaction was then quenched with EDTA (0.3 M final concentration) by mixing in the collection tube. The decreasing amount of product formed in the rapid reaction with UTP provides a direct measurement of the decreasing amount enzyme-RNA complex as a function of time during mixing step 1 with the heparin trap (Figure 3E). The dissociation rate is slow, so the best fit obtained by simulation gives an RNA dissociation rate constant of 0.013 ± 0.001 s^−1^ (*k*_*off*_ in the Scheme in Figure 3). Confidence contours for the RNA off rate and UTP incorporation rate constants are shown in Figures 3G and 3H, respectively.

To obtain a minimal estimate of the active fraction of enzyme, we locked the RNA dissociation rate at 0.013 s^−1^ and fit the enzyme titration experiment in Figure 3D and UTP incorporation data in Figures 3A–3C using KinTek Explorer using the scheme shown at the top of Figure 3. The experiments were modeled with a pre-equilibration of the E and X states, where E is the active enzyme and X is inactive enzyme. This is not to say that enzyme equilibrates between active and inactive state; rather, this model provides a method to estimate the fraction of enzyme that is active. Equilibration between the E and X states was made very slow (10^−5^ s^−1^) and allowed to reach equilibrium (computationally for ~6 days) before adding RNA in a separate step and then nucleotide in a final step, both of which occur on timescales fast enough to not perturb the E to X equilibrium.

Confidence contours for *K*_*active*_, and the resulting *k*_*on*_ are shown in Figure 3F. This data fitting shows that the data define a lower limit of 60% active enzyme and that the resulting *K*_*d*_ (from varying *k*_*on*_) changes based on the active fraction. At 60% active enzyme, the resulting *K*_*d*_ for RNA binding is 1.3 μM, whereas at 100% active enzyme the resulting *K*_*d*_ would be 2.2 μM, giving the range of possible *K*_*d*_ values from the data. The RNA dissociation rate does not vary significantly over this range because it is well defined by the data in Figure 3E and the interpretation is independent of the fraction of active enzyme over the limited range (60%–100%). The linear relationship between calculated binding rate constant and fraction of active enzyme reflects the fact that the net RNA binding rate with enzyme in excess is defined by the product of *k*_*on*_[E]. The apparent RNA binding rate constants are far below diffusion limits (0.008 μM^−1^s^−1^ calculated for 80% active enzyme), which could be attributed to a two-step binding mechanism; however, the experiments presented here cannot differentiate multiple binding steps, so the simple one-step binding model was used.

Globally fitting the data shown in Figures 3A–3C was finalized assuming that 80% of the enzyme was active and with RNA binding kinetics defined from Figures 3D and 3E. The *k*_*cat*_ and *K*_*m*_ values for the first and second UTP incorporations are summarized in Table 1. These parameters do not significantly vary over the range of 60%-100% active enzyme (Table S1).

### Remdesivir Is Incorporated More Efficiently Than ATP by the SARS-CoV-2 RdRp Complex

**Figure 4:**
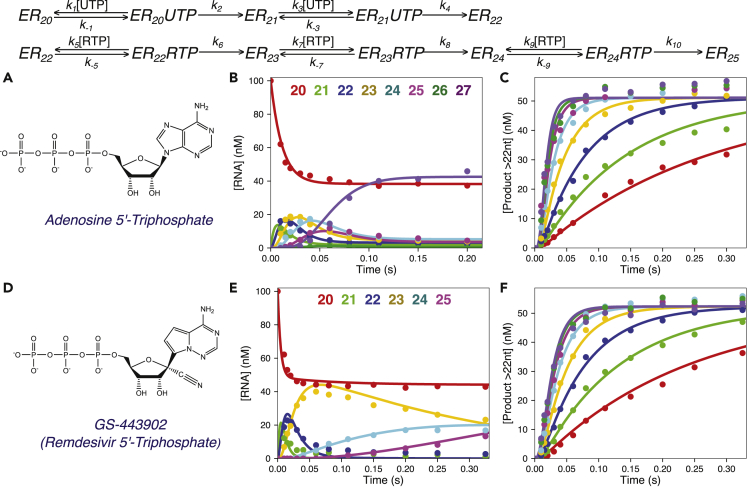
Remdesivir Is Incorporated more Efficiently than ATP by the SARS-CoV-2 RdRp Complex Scheme: kinetic pathway for UTP and ATP incorporation. Species are labeled as in Figure 2, with added steps for sequential RTP incorporations; the same sequence applies to ATP incorporation. Net rate constants *k*_*5*_, *k*_*7*_, and *k*_*9*_ define *k*_*cat*_/*K*_*m*_ for each sequential RTP incorporation, whereas *k*_*6*_, *k*_*8*_, and *k*_*10*_ define *k*_*cat*_ for each sequential RTP incorporations. For ATP the pathway is identical, but we fit the data to only define *k-*_*5*_ and *k*_*6*_ defining the kinetic parameters for the first incorporation, modeled as the sum of all products ≥23 nt in length. (A) Chemical structure of adenosine 5′-triphosphate. Experiments: a solution containing 1.25 μM NSP12/7/8 complex, 6 μM NSP8, 100 nM FAM-20/40 RNA, and 5 mM Mg^2+^ was mixed with 200 μM UTP and varying concentrations of ATP (5–400 μM) to start the reaction, and products were resolved and quantified using capillary electrophoresis. (B) Reaction progress curve for individual RNA species at 225 μM ATP. Products of primer extension from 20 to 27 nt in length are shown as a function time after mixing. Note the reaction is over in less than 0.2 s. The solid lines through the data points show the best global fit by simulation for each RNA species with an average rate of ATP incorporation around 80 s^−1^ at this concentration. The color code for each RNA species is given at the top of the figure. (C) Concentration of ATP incorporation product versus time at various ATP concentrations. We plot the sum of all products after the incorporation of the first ATP, i.e., products 23–27 nt in length to define the kinetics of the first ATP incorporation. The solid lines show the best global fit by simulation for the total RNA product ≥23 nt at each ATP concentration, shown as different colors (red to purple: 5, 10, 20, 40, 80, 150, 225, and 400 μM). These data define the parameters *k*_*cat*_ and *K*_*m*_ for ATP incorporation as shown in Table 1. (D) Chemical structure of GS-443902 (Remdesivir 5′-triphosphate, RTP). RTP is an adenosine analog with a 1′ cyano group and modifications to the adenine base. Note that Remdesivir contains a 3′-OH, allowing continued polymerization after its incorporation. A mixture containing 1.5 μM NSP12/7/8 complex, 6 μM NSP8, 100 nM FAM-20/40 RNA, and 5 mM Mg^2+^ was mixed with 150 μM UTP and varying concentrations of RTP (3.5–315 μM) to start the reaction in the quench-flow instrument. Reactions were quenched with EDTA after various reaction times and the products quantified by capillary electrophoresis. (E) Concentration of each RNA species versus time at 315 μM RTP. The concentrations of various species, 20 nt (red), 21 nt (green), 22 nt (blue), 23 nt (yellow), 24 nt (cyan), and 25 nt (purple) are shown with the solid lines from the best global fit of the data by simulation in KinTek Explorer, which included the time course for each species at each RTP concentration (not shown). (F) Concentration of product containing RTP versus time for various concentrations of RTP. The time dependence of the first RTP incorporation is shown as the sum of species ≥23 nt in length. The best fit by simulation is shown as the solid colored lines through the data points, with different colors for each RTP concentration (red to purple: 3.5, 7, 14, 28, 56, 112, 210, and 315 μM). The parameters *k*_*cat*_*and* K_m_ for each Remdesivir incorporation were derived by globally fitting all of the primary data defining the formation and decay of products 23, 24, and 25 nt in length and are summarized in Table 1.

Because Remdesivir (Figure 4D) is an ATP (Figure 4A) analog, we first measured the kinetics of ATP incorporation by rapid quench methods so the specificity constant (*k*_*cat*_/*K*_*m*_) for ATP provides a benchmark for evaluating the relative efficiency of Remdesivir incorporation. A solution of 1.25 μM NSP12/7/8 complex, 6 μM NSP8, 100 nM FAM-20/40 RNA, and 5 mM Mg^2+^ was mixed with 200 μM UTP and varying concentrations of ATP (5–400 μM) to start the reaction. The RNA substrate used in these studies encodes for two UTPs followed by four ATPs and then another UTP to form the 27 nt product. The data for each product band at 225 μM ATP are shown in Figure 4B showing the rise and fall of each intermediate.

Because the rates for each ATP incorporation appear similar and rapid after UTP incorporation, the kinetic parameters for each individual ATP incorporation were not well resolved in globally fitting the data (see scheme in Figure 4). Therefore, products ≥23 nt, corresponding to the products with ≥ one ATP incorporated, were summed to give the kinetics of incorporation of the first ATP (defined by *K*_*5*_ and *k*_*6*_). The data for the total product ≥23 nt at each ATP concentration are shown in Figure 4C. Fitting the data with known kinetics for incorporation of the two UTPs affords accurate estimates of the kinetic parameters governing the first ATP incorporation: *K*_*m*_ = (*k*_*-5*_+*k*_*6*_)/*k*_*5*_ and *k*_*cat*_ = *k*_*6*_ (Table 1). In particular, *k*_*cat*_/*K*_*m*_ represents the most important parameter to evaluate Remdesivir incorporation because the ratio of *k*_*cat*_/*K*_*m*_ values for the two nucleotides defines the ability of the enzyme to discriminate against Remdesivir in favor of ATP, as described below.

To investigate the kinetics of Remdesivir triphosphate (RTP) incorporation by the SARS-CoV-2 NSP12/7/8 complex, the concentration dependence of Remdesivir on the kinetics of its incorporation were measured by rapid quench methods, as described for ATP. A solution of 1.5 μM NSP12/7/8 complex, 6 μM NSP8, 100 nM FAM-20/40 RNA, and 5 mM Mg^2+^ was equilibrated then mixed with 150 μM UTP and various concentrations of RTP (3.5–315 μM) to start the reaction. Data at 315 μM RTP are shown in Figure 4E to illustrate the time dependence for formation and decay of each species. As with studies on ATP incorporation, because the kinetics of UTP incorporation are known (*k*_*1*_ through *k*_*4*_ in the scheme in Figure 4), the incorporation of Remdesivir can be easily modeled and fit by simulation. After the two templating A's, there are 4 templating U's followed by another templating A, giving 4 opportunities for RTP incorporation followed by the opportunity for one UTP incorporation. The rate constants for the first RTP were obtained by summing all of the products ≥23 nt in length as shown in Figure 4F. Fitting the data by simulation shows that the first RTP was efficiently incorporated: *k*_*cat*_*/K*_*m*_ = 1.29 ± 0.06 μM^−1^s^−1^, *k*_*cat*_ = *k*_*6*_ = 68 ± 2 s^−1^, and *K*_*m*_ = 53 ± 2 μM (Table 1). Fitting the complete time course for each species (exemplified by one RTP concentration in Figure 4E) afforded resolution of all three incorporation reactions. Incorporations of the second and third RTP (*k*_*7*_ through *k*_*10*_ in the scheme in Figure 4) occur with lower efficiencies with *k*_*cat*_*/K*_*m*_ values (~0.19 μM^−1^s^−1^) and slower *k*_*cat*_ (3.6–3.8 s^−1^) leading to lower *K*_*m*_ values (Table 1). The slower rates for the second and third incorporation allow the kinetics to be resolved in global data fitting. Even though the *k*_*cat*_ values are lower than for ATP, the data demonstrate that at least 3 RTPs are incorporated efficiently.

**Figure 5:**
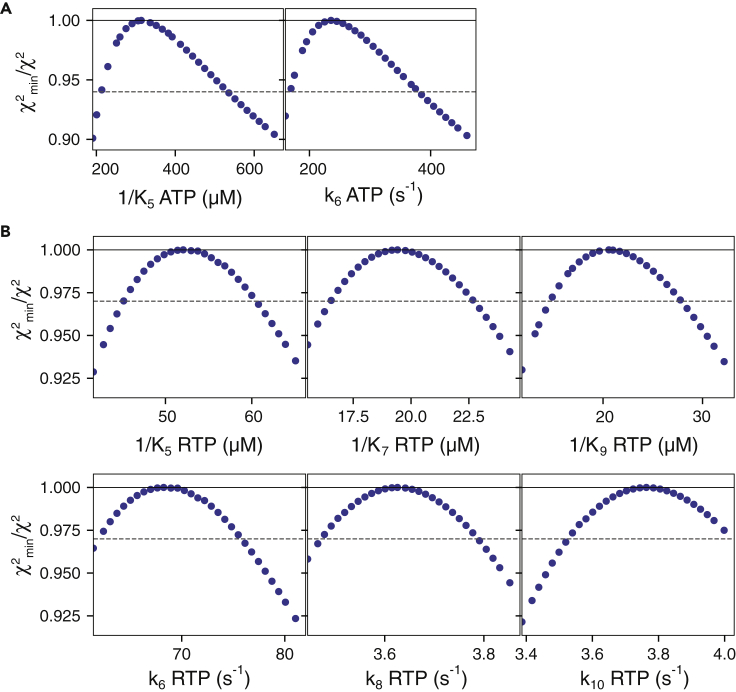
Confidence Contour Analysis (A) Confidence contours for ATP incorporation. Confidence contours were derived from fitting the ATP incorporation data to determine 1/*K*_*5,ATP*_ and *k*_*6,ATP*_. The data are slightly skewed, with a well-defined lower limit but a less well-defined upper limit due to the two sequential UTP incorporations that occur before ATP incorporation. The error estimates were based on a χ^2^ threshold of 0.94 for the ratio of χ^2^_min_/χ^2^ (dashed line) to define the 95% confidence limits (Johnson, 2019; Johnson et al., 2009a). (B) Confidence contours for RTP incorporation. Confidence contours were derived from fitting the RTP incorporation data to determine 1/*K*_*5,RTP*_, 1/*K*_*7,RTP*_, 1/*K*_*9,RTP*_, *k*_*6,RTP*_, *k*_*8,RTP*_, and *k*_*10,RTP*_. Based on the number of parameters and data points, the threshold was 0.97 in fitting the RTP incorporation data to define the 95% confidence limits. Values for UTP incorporation were locked at the values given in Table 1 during the fitting. Rate constants are defined according to the scheme in Figure 4. Error estimates listed in Table 1 are based on the standard errors derived during nonlinear regression analysis. The estimated errors are consistent with the confidence intervals derived from confidence contour analysis, noting that the confidence interval is 1.96 times the standard error range.

Confidence contours in Figure 5 show that each parameter is well constrained by the data. Error estimates in Table 1 were derived from the standard errors derived during nonlinear regression analysis. Such error estimates can be misleading if the parameters are not well constrained by the data. In the present case, the symmetrical confidence contour analysis establish that each parameter is well constrained and therefore, the standard error estimates are valid (Johnson, 2019). Multiplying the standard error by 1.96 gives the 95% confidence interval similar to values derived by confidence contour analysis. Incorporation of a fourth RTP occurs slowly, with only a small amount observed at the highest concentration. On the timescale of the experiment, no incorporation of UTP was observed after the four molecules of Remdesivir, but it may occur after longer times of incubation, especially due to the absence of the proofreading exonuclease in this experiment so each incorporation reaction is largely irreversible.

## Discussion

As of October 25, 2020, the World Health Organization reports over 43 million confirmed cases and over 1.15 million deaths due to SARS-CoV-2 globally with new infections increasing by 33% in a last week (WHO, 2019). Although development of a vaccine for SARS CoV-2 appears promising, it will still be important to develop an arsenal of direct-acting antiviral drugs for individuals who fail to develop neutralizing antibodies or neglect to get vaccinated. The viral RdRp is an attractive target for direct-acting antiviral drugs. In particular, nucleoside analogs may be effective in treating newly arising coronaviruses due to conservation of active site residues needed for activity. For example, nucleoside analogs and nonnucleoside inhibitors of the HIV reverse transcriptase are the cornerstone of treatments to control HIV infections (Skowron and Odgen, 2006). Similarly, HCV infections are effectively treated by the nucleoside analog, Sofosbuvir, which targets the viral RdRp. On October 22, 2020 Remdesivir became the first drug to be approved by the FDA for treating COVID-19 (FDA, 2020).

We found that Remdesivir is incorporated by the RdRp complex more efficiently than ATP, defined by the relative *k*_*cat*_*/K*_*m*_ values for RTP versus ATP (1.29 versus 0.74 μM^−1^s^−1^). Although RTP is incorporated at least three-fold slower than ATP (68 s^−1^ versus 240 s^−1^), it has a six-fold higher *k*_*cat*_*/K*_*m*_ value due to a six-fold lower *K*_*m*_ relative to ATP (53 μM versus 320 μM). We define a *discrimination index* as the ratio of specificity constants for the normal nucleotide divided by that for the analog—this term quantifies the ratio by which the enzyme discriminates against the nucleotide analog (AnTP) relative to the normal nucleotide (NTP).

Discrimination index = *D* = (*k*_*cat*_*/K*_*m*_)_NTP_/(*k*_*cat*_*/K*_*m*_)_AnTP_

Nucleotides incorporated per analog = *D* x [NTP]/[AnTP]

The value of the discrimination index for RTP versus ATP is less than one (0.57), which indicates that the analog is a better substrate than the cognate nucleotide. Of the nine nucleoside analogs approved by the FDA to treat HIV infections, only Stavudine (a thymidine analog) has a discrimination index less than one, but it has a higher toxicity index due to incorporation by the human mitochondrial DNA polymerase (Johnson et al., 2001). Certainly, the discrimination index of 0.57 for RTP will help it to compete with ATP, but its effectiveness may still be limited by the high concentration ATP in the cell, typically around 3 mM (Traut, 1994). For example, an intracellular RTP concentration of 10 μM will lead to incorporation of RTP only one time out of 170 uridines in the template. However, this may be sufficient. For example, Sofosbuvir is effective in treating HCV infections even though it is incorporated inefficiently with a *k*_*cat*_*/K*_*m*_ = 0.0007 μM^−1^s^−1^ compared with 0.1 μM^−1^s^−1^ for UTP, giving a discrimination index = 140 ± 45 (Villalba et al., 2020). The physiological concentration UTP ranges from 110 to 1000 μM UTP (Traut, 1994), so given a hypothetical concentration of 10 μM Sofosbuvir-triphosphate, Sofosbuvir will be incorporated once out of 1500–14,000 opportunities. In evaluating both Remdesivir and Sofosbuvir, we do not know the physiological concentrations of the activated, triphosphate form of the nucleoside analogs, so our calculations of relative effectiveness assume the same nominal concentration (10 μM) for comparison. A higher concentration of the triphosphate form would make the analogs more effective, so it would be important to obtain estimates of the physiological concentrations of the activated form of each analog. Nonetheless, this analysis explains why Remdesivir is effective in spite of having to compete against a high concentration of ATP.

We also discovered that further addition of RTP on top of an incorporated RTP proceeds with *k*_*cat*_*/K*_*m*_ values lower than that for the first RTP mostly because the rate constants for incorporation (*k*_*cat*_) were 18-fold slower (Table 1). Nonetheless, it is unlikely that the RdRp would incorporate multiple sequential RTPs *in vivo*. If the probability of incorporating one RTP is 1/170 = 0.6%, then the probability of incorporating two RTPs would be 0.004%. However, we do not know the rate of any NTP incorporation after RTP, and the unusual structure of Remdesivir could alter the kinetics of the incorporation of subsequent nucleotides. It has been suggested that Remdesivir may be a delayed chain terminator (Gordon et al., 2020b) but that hypothesis needs to be tested more rigorously in studies including the proofreading exonuclease to establish the balance between incorporation versus excision.

The major unanswered question is the extent to which incorporated Remdesivir is resistant to excision by the proofreading exonuclease, NSP14/10 (Ferron et al., 2018; Ma et al., 2015). For example, Sofosbuvir is more effective *in vivo* than a similar analog, Mericitabine, because it escapes removal by a highly active ATP-dependent excision reaction catalyzed by the HCV RdRp. Although Mericitabine is incorporated 40-fold more efficiently than Sofosbuvir, it has a half-life of less than one minute after incorporation, but Sofosbuvir persists with a half-life of at least one day (Villalba et al., 2020). Although inefficiently incorporated, Sofosbuvir resists excision. In contrast, RTP is more efficiently incorporated at physiological ATP concentrations than Sofosbuvir is at physiological UTP concentrations, but the question as to the resistance to exonuclease removal of RMP remains to be addressed quantitatively. It appears that resistance to exonuclease hydrolysis will be the predominant determinant of the effectiveness of nucleoside analogs in combating coronavirus infections (Robson et al., 2020). More work is needed to define the kinetic and structural features governing the exonuclease activity and selectivity.

Two criteria must be met when optimizing the expression and purification of a polymerase. The first essential requirement is that the enzyme must catalyze polymerization with rates commensurate with the time estimated for replication *in vivo*. The second criterion is based on measurement of the fraction of enzyme that is active, often addressed by an active site titration. In our studies, average rates of polymerization of 300 base pairs per second are sufficient to replicate the coronavirus genome in less than 2 min. There is sufficient information within the available data to show that our enzyme is at least 60% active relative to the nominal concentration determined by absorbance measurements. This estimate is based on confidence contour analysis demonstrating that a good fit to the data could not be obtained if the active enzyme was less than 60% of the nominal concentration. However, our equilibrium titration (Figure 3D) shows that the RNA binding affinity is weak (1.8 ± 0.2 μM). It is quite likely that the addition of the exonuclease subunits (NSP14/10) will lead to tighter RNA binding, which will afford a more accurate measurement of the active site concentration.

Although tagged protein expression in insect cells has many advantages and has proven to be an effective platform for expression of the SARS-CoV-2 RdRp for *in vitro* studies, the expense and time required for protein production are major limitations. The use of tags to aid in purification also have their own set of limitations and often alter the activity of enzymes. We initially tested expression of untagged NSP12 in *E. coli* and found the protein was almost totally insoluble under a wide range of conditions; however, this was almost completely alleviated by co-expression of NSP12 with NSP7 and NSP8 along with the chaperones Tf, GroEL/ES. The large quantity of soluble protein expressed in *E. coli* greatly facilitated our search for conditions to optimize efficient purification of the tag-free enzyme. We also found the untagged enzyme to have higher activity than the 8xhistidine-tagged NSP12/NSP7L8 construct used in previous studies (Shannon et al., 2020). The lower activity could be due to either the histidine tag, the linked NSP7L8, or both. However, we have not repeated these results nor done an extensive characterization of the tagged enzyme. Therefore, our results only raise a caution in using tagged enzymes.

Previous studies on the coronavirus RdRp have mostly relied on steady-state methods that are unreliable except for comparison of two competing substrates when both are present in the reaction mixture. As might be expected, *K*_*m*_ estimates from steady state are around 1,000-fold lower when comparing our results to prior publications (Gordon et al., 2020b; Shannon et al., 2020). It has long been known from assays of DNA polymerase kinetics that steady-state measurements underestimate *k*_*cat*_ and *K*_*m*_ by factors of 100- to 1000-fold due to the rate-limiting dissociation of the enzyme-DNA complex in the steady state (Johnson, 2010). Prior steady-state kinetic studies on the coronavirus RdRp have reported *k*_*cat*_ values that rely on units of velocity as fraction of primer extended per minute that are meaningless (Gordon et al., 2020a, 2020b). These errors are attributable to a relatively inactive enzyme preparation and faulty experimental design. Robust assays must be employed so that the experimentalist knows which step in the reaction limits the observed output for studies evaluating the kinetics and mechanism of inhibition. This is especially important for high-throughput screens. For example, inhibitors that slow the steady-state rate of RNA dissociation may not be very effective *in vivo*. More importantly, the effects of inhibitors that slow the rates of incorporation will be masked by the slow RNA dissociation rate when measurements are made in the steady state.

Our yield of active, untagged protein allowed us to perform pre-steady-state experiments with the enzyme concentration in large excess over the RNA to observe polymerization during a single turnover. This is the key for obtaining reliable and quantitative definition of the kinetics of each single nucleotide incorporation event during processive synthesis. Measurements under pre-steady-state conditions provides accurate estimates of *k*_*cat*_ and *K*_*m*_ for each single nucleotide incorporation event during processive polymerization. Traditionally, the terms *k*_*pol*_ and *K*_*d,app*_ have been used to define parameters derived from pre-steady-state DNA polymerase kinetics. The initial intention was to distinguish the results from faulty steady-state analysis and to indicate that if nucleotide binding occurs in one step, then *K*_*d,app*_ is a true *K*_*d*_ (Johnson, 1993). However, in general, the *K*_*d,app*_ = *K*_*m*_ for each incorporation during processive synthesis even when nucleotide binding occurs in more than one step (Kellinger and Johnson, 2010). Our analysis will allow for accurate comparison of binding and incorporation parameters for nucleoside analogs relative to their natural counterparts in the search for new inhibitors.

### Limitations of the Study

Although nucleotide incorporation kinetics are important for developing analogs that efficiently compete with natural nucleotides for incorporation, this is only half of the story. The SARS-CoV-2 has a proofreading exonuclease complex consisting of NSP10/14 (Bouvet et al., 2012; Ma et al., 2015) that can likely excise nucleotide analogs once incorporated into the RNA, thus preventing chain termination and allowing the virus to continue replication. Moreover, studies performed in the absence of the proofreading exonuclease (NSP14/10) do not address the fundamental questions as to whether Remdesivir resists excision. In the absence of the exonuclease, continued polymerization will eventually bury Remdesivir. On the other hand, it is conceivable that after incorporation of several nucleotides on top of Remdesivir, the terminal nucleotide will be excised by the exonuclease and then reinserted by the polymerase in a repeated cycle of addition and excision to reach a steady state. Future studies on nucleotide analog excision by the NSP10/14 proofreading exonuclease complex, using pre-steady-state methods, will be crucial to analyze in conjunction with the polymerization data to discover the most useful antivirals for treating SARS-CoV-2 infection.

### Resource Availability

#### Lead Contact

Questions about or requests for materials should be directed to and will be fulfilled by the Lead Contact, Kenneth A. Johnson (kajohnson@mail.utexas.edu).

#### Materials Availability

Plasmids used in this study have been deposited to Addgene: pcI^ts,ind+^-(NSP8) – 160656 pQE-(NSP12)-pcI^ts,ind+^-(NSP7-NSP8) – 160540.

#### Data and Code Availability

The published article includes all datasets generated or analyzed during this study.

## Methods

All methods can be found in the accompanying Transparent Methods supplemental file.

## Supplemental Information

Document S1. Transparent Methods, Figures S1–S6, and Table S1
