# Supplementary material for: Remdesivir Is Effective in Combating COVID-19 because It Is a Better Substrate than ATP for the Viral RNA-Dependent RNA Polymerase
Source: iScience. 2020 Nov 28;23(12):101849. doi: 10.1016/j.isci.2020.101849 (PMC7695572; doi:10.1016/j.isci.2020.101849)
Supplement: Document S1. Transparent Methods, Figures S1–S6, and Table S1 [file mmc1.pdf]

iScience, Volume *23*

Supplemental Information

Remdesivir Is Effective in Combating COVID-19 because It Is a Better Substrate than ATP for the Viral RNA-Dependent RNA Polymerase

Tyler L. Dangerfield, Nathan Z. Huang, and Kenneth A. Johnson

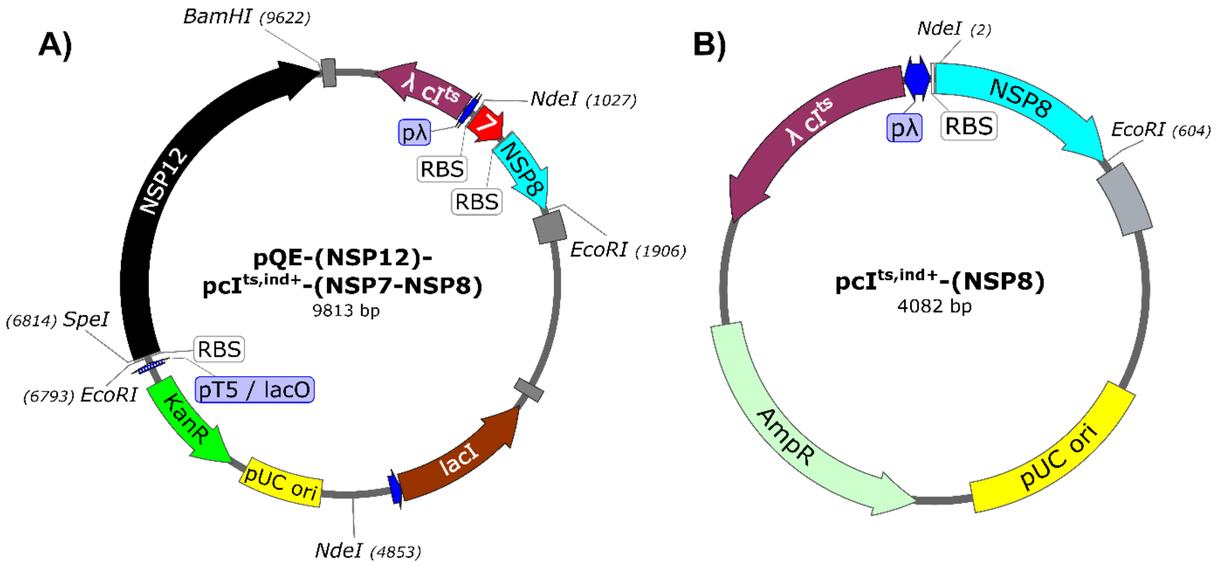

<u>Figure S1: Expression plasmids, related to Figure 1.</u> These maps show the plasmids that were used for *E. coli* expression of SARS-CoV-2 replication complex genes. Restriction sites are shown on the outside of the plasmid while unique features are shown on the plasmid or on the inside of the plasmid. <u>A) Plasmid for NSP12/8/7 co-expression.</u> This plasmid contains the NSP12 gene (black) under the T5 promoter and lac operator (blue, pT5/lacO), controlled by the on-board lacI gene (brown) under the placI promoter (blue) and induced with addition of IPTG. The NSP7 (7, red) and NSP8 (cyan) genes are arranged in a bicistronic operon, with ribosome binding sites (RBS, white) upstream of each gene—expression is under control of the lambda promoter system. The plasmid also features the kanamycin resistance gene (KanR, green) and a high-copy number pUC origin of replication (pUC ori, yellow). <u>B) NSP8 expression plasmid.</u> The plasmid shown was used for NSP8 (cyan) expression in *E. coli* under control of the lambda promoter (pλ, blue) and *E. coli* $rrnBT_1T_2$ terminator (grey) with an on-board temperature sensitive, recA cleavable $cI^{ts,ind+}$ repressor variant (λ $cI^{ts,ind+}$, purple). This plasmid also features a pUC origin of replication (pUC ori, yellow) and an ampicillin resistance gene (AmpR, green). Expression of NSP8 was induced by shifting the culture temperature from 30°C to 42°C for 30 minutes, followed by 3 hours of expression at 38°C.

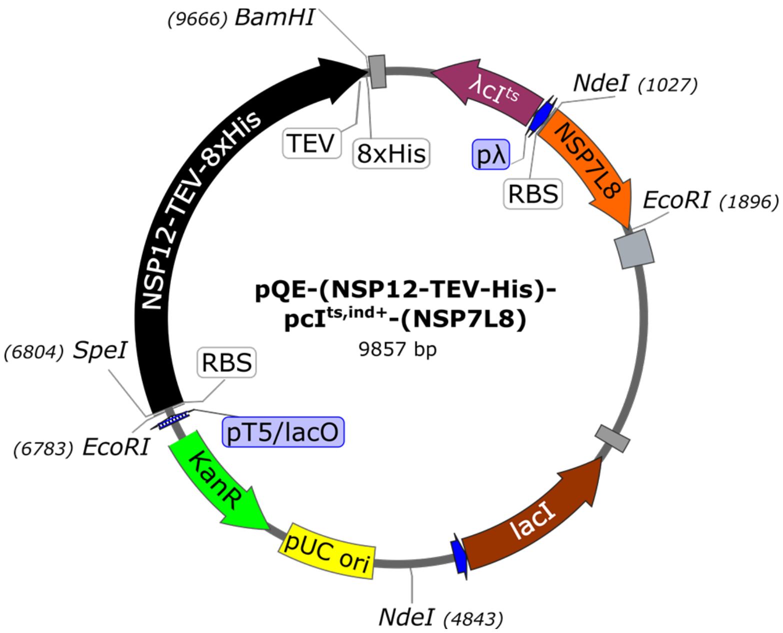

Figure S2: Histidine tagged NSP12/NSP7L8 expression plasmid, related to Figure 1. Contains the NSP12-TEV-8xHis gene (black) cloned under the T5 promoter/lac operator (pT5/lacO, blue), as well as the NSP7L8 gene (orange) containing a GSGSGS linker between NSP7 and NSP8 under control of the lambda promoter (pλ, blue). This plasmid also contains the lacI gene (brown) and the λcI$^{ts,ind+}$ repressor (purple) so expression can be performed in most *E. coli* strains. The kanamycin resistance gene (KanR, green) for selection and pUC origin of replication (yellow) are also on the plasmid.

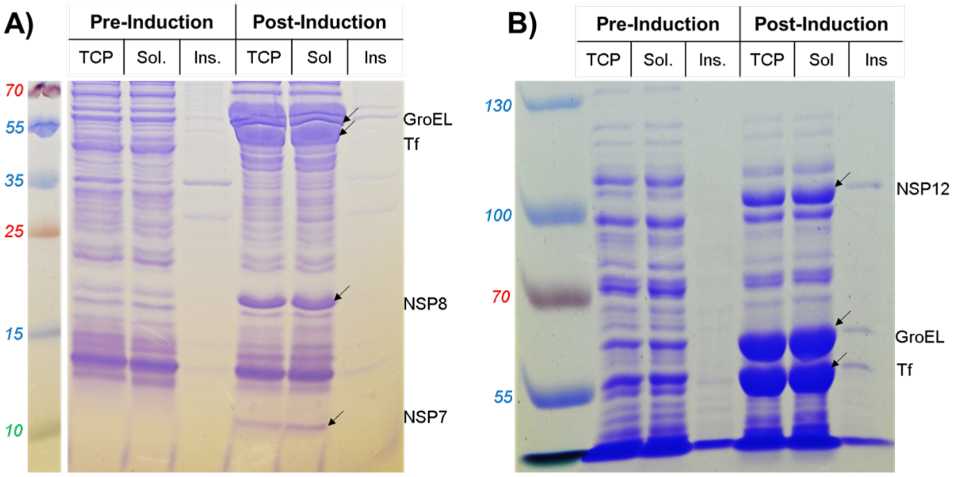

Figure S3: Soluble expression of NSP12/7/8 complex, related to Figure 1. Molecular weights of the protein ladder in kDa are given to the left of each gel in the color of its corresponding band. Samples corresponding to the total cell protein fraction (TCP), the soluble protein fraction (Sol.), and the insoluble protein fraction (Ins.) were from samples of *E. coli* cells before induction (Pre-Induction) and at the time of harvesting the pellet (Post-Induction). Molecular weights of proteins of interest are: NSP7, 9.4 kDa; NSP8, 22 kDa; NSP12, 107 kDa; Tf, 56 kDa; GroEL, 60 kDa. A) 15% SDS-PAGE of RdRp complex expression. The proteins of interest are labeled to the right of the gel with arrows pointing to the relevant bands B) 6% SDS-PAGE of RdRp complex expression. Proteins of interest are labeled as in (A).

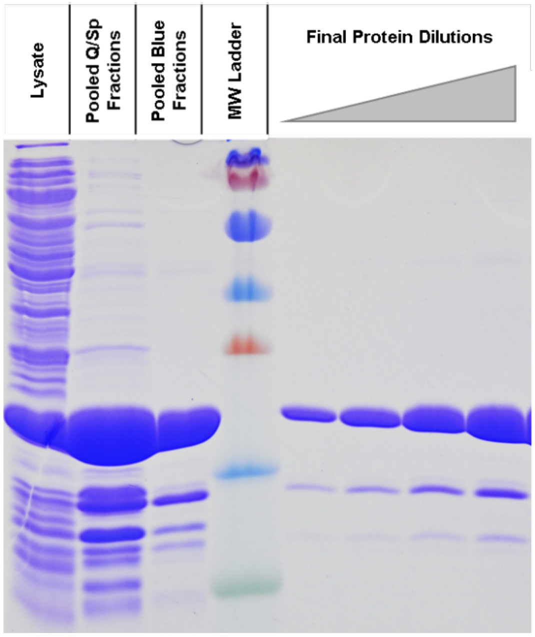

Figure S4: Expression and purification of NSP8, related to Figure 1. Bands in the molecular weight ladder are as follows in kDa in order from the bottom green band to the top blue band: 10, 15, 25, 35, 55, 70, 100. The leftmost lane contains a sample of the clarified lysate (soluble fraction) before loading on the Q/SP columns. The next two lanes to the right are samples collected after the Q/SP columns and after the HiTrap Blue-FF column. The lanes to the right of the molecular weight ladder are dilutions of the final protein, after the size exclusion column.

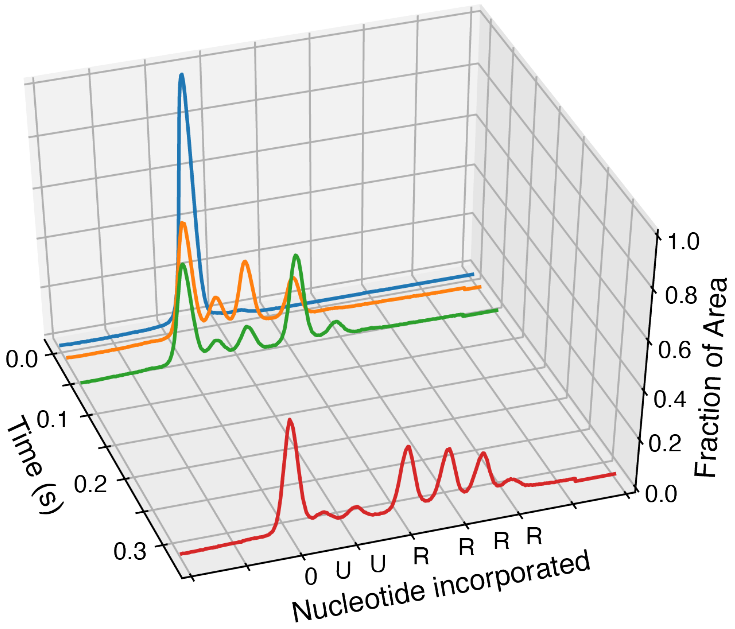

Figure S5: Sample chromatograms for Remdesivir incorporation experiment, related to Figure 4. A 3-D plot of chromatograms from different time points is shown with reaction time on the x axis, nucleotide position (retention

time relative to internal standard) on the y axis, and fractional intensity on the z axis. Peaks at each time point are sufficiently resolved and there is no indication of re-annealing of the RNA during electrophoresis.

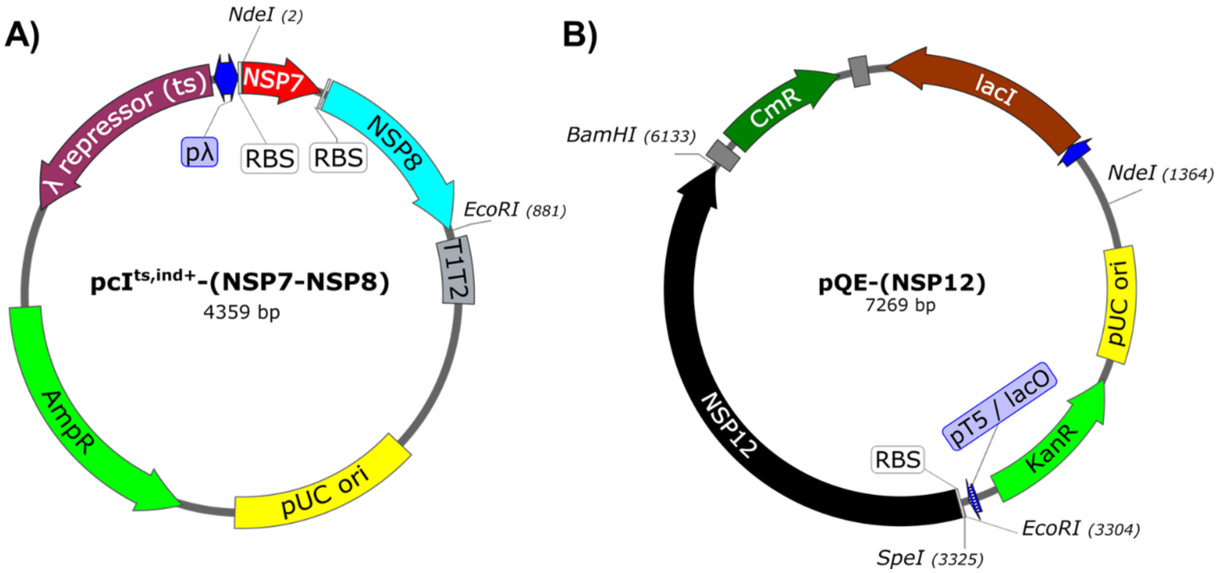

<u>Figure S6: Precursor expression plasmids, related to Figure 1.</u> <u>A) Bicistronic NSP7/NSP8 expression plasmid.</u> This plasmid contains the bicistronic NSP7-NSP8 operon cloned under the lambda promoter (pλ, blue) and *E. coli* rrnBT_1T_2 terminator (T1T2, grey), used with pQE-(NSP12) to make the final expression plasmid for the replication complex. The bicistronic operon contains ribosome binding sites (RBS, white) upstream of both NSP7 (red) and NSP8 (cyan) genes and a short linker between the stop codon for NSP7 and the RBS for NSP8. This plasmid also contains the temperature or nalidixic acid inducible λ$cI^{ts,ind+}$ repressor (λ repressor, purple) cloned on the plasmid so expression can be performed in most *E. coli* strains. It also contains the ampicillin resistance gene (AmpR, green) for selection and the high copy number pUC origin of replication. Restriction sites of interest are given on the outside of the plasmid. <u>B) NSP12 expression plasmid.</u> This plasmid contains the codon optimized NSP12 gene (black) under control of the T5 promoter/lac operator (pT5/lacO, blue), used for constructing the final expression plasmid for co-expression with NSP7 and NSP8. This plasmid also features a pUC origin of replication (yellow), the chloramphenicol resistance gene (CmR, dark green), the kanamycin resistance gene (KanR, light green), and lacI repressor gene (brown). Restriction sites of interest are given on the outside of the plasmid map.

Table S1. Comparing 64% and 80% active enzyme, related to Figure 3

Parameter	64% active	80% active
k_{-1} (s^{-1})	10800 ± 800	10800 ± 800
k_2 (s^{-1})	370 ± 20	370 ± 20
k_{-3} (s^{-1})	17000 ± 3000	17000 ± 3000
k_4 (s^{-1})	380 ± 50	380 ± 50
k_{off} (s^{-1})	0.0132 ± 0.001	0.0142 ± 0.001

Rate constants and standard errors derived in fitting shown in Figure 3, where k_1 and k_3 were locked at 100 μM^{-1} s^{-1}.

Transparent Methods

METHOD DETAILS

Cloning:

All restriction enzymes, T4 ligase, T4 DNA polymerase, and Phusion DNA polymerase were purchased from New England Biolabs. Oligonucleotides used for cloning were synthesized by Integrated DNA Technologies with standard desalting and used without further purification. Plasmid figures were prepared with SnapGene® software (from GSL Biotech; available at snapgene.com). Genes were synthesized with codon optimization for *E. coli* by GenScript in pUC57 backbones. NSP12 was synthesized with an *NdeI* site integrated into the start codon and an *EcoRI* site at the 3’ end of the gene, and the plasmid containing the gene was designated pUC57-(NSP12). Only the methionine from the start codon (ATG) was added to the wild-type NSP12 gene. NSP7 and NSP8 genes were synthesized as a bicistronic operon with an *NdeI* site integrated into the NSP7 start codon, a short linker region containing a ribosome binding site between the stop codon for NSP7 and the start codon for NSP8, and an *EcoRI* site at the 3’ end of the NSP8 gene. The plasmid for cloning with the bicistronic operon was designated pUC57-(NSP7-NSP8). The bicistronic NSP7-NSP8 operon was cloned from pUC57-(NSP7-NSP8) into the $pcI^{ts,ind+}$ backbone (Brandis and Johnson, 2009) under the λ promoter, using *NdeI* and *EcoRI* restriction sites to yield $pcI^{ts,ind+}$-(NSP7-NSP8) shown in Figure S6A. The plasmid $pcI^{ts,ind+}$-(NSP8) in Figure S1B was made by PCR from $pcI^{ts,ind+}$-(NSP7-NSP8), using primers to delete the NSP7 gene. The NSP12 gene was cloned from pUC57-(NSP12) into the pQE-30 backbone with *SpeI* and *BamHI* sites, added by PCR, at the 5’ and 3’ ends of the NSP12 gene, respectively, to yield pQE-(NSP12) shown in Figure S6B. Ligation independent cloning (Aslanidis and de Jong, 1990) was used to join amplicons from $pcI^{ts,ind+}$-(NSP7-NSP8) and pQE-(NSP12), to form pQE-(NSP12)-$pcI^{ts,ind+}$-(NSP7-NSP8) shown in Figure S1A. The ampicillin resistance and chloramphenicol resistance genes were removed during this step, leaving only the kanamycin resistance gene on the final plasmid.

The following steps were performed to make the histidine-tagged NSP12 complex/NSP7L8 complex: The plasmid $pcI^{ts,ind+}$-(NSP7-NSP8) was amplified by PCR, using primers to replace the linker region between NSP7 and NSP8 with a GSGSGS linker, to yield $pcI^{ts,ind+}$-(NSP7L8). The plasmid pQE-(NSP12) was amplified by PCR using primers to add the following amino acid sequence to the C terminus of NSP12, as previously reported (Shannon et al., 2020): GGSENLYFQGHHHHHHHH, consisting of a 3 amino acid linker, a TEV protease site, and 8 histidine residues. The resulting plasmid was designated pQE-(NSP12-TEV-8xHis). A fragment of pQE-(NSP12-TEV-8xHis) and a fragment from $pcI^{ts,ind+}$-(NSP7L8) were amplified by PCR and joined with ligation independent cloning, as above, to yield pQE-(NSP12-TEV-8xHis)-$pcI^{ts,ind+}$-(NSP7L8) shown in Figure S2. pG-Tf2 (Nishihara et al., 2000) was purchased from Takara Biosciences. All constructs were verified by Sanger sequencing, which was performed by the University of Texas at Austin sequencing facility.

Protein expression:

Large scale expression protocols are given below for each construct. Samples for the total cell protein, soluble and insoluble fractions for gel electrophoresis analysis shown in the supplemental information were prepared as previously described (Brandis and Johnson, 2009).

NSP8 expression:
BL21 *E. coli* harboring pcI$^{ts,ind+}$-(NSP8) were inoculated into Terrific Broth (2.4% yeast extract, 2% tryptone, 0.4% glycerol, 17 mM KH_2PO_4, 72 mM K_2HPO_4) with 100 μg/ml ampicillin and grown overnight at 30°C with shaking. The overnight starter culture (8 ml) was used to inoculate 1 liter of Terrific Broth with ampicillin in a 2.8 liter baffle-bottomed flask. Cells were grown at 30°C with shaking at 250 rpm until the OD_{600} reached 2. NSP8 expression was induced by incubation of the cells at 42°C with shaking for 20 minutes, then the cultures were incubated for an additional 3 hours with shaking at 38°C. Cells were harvested by centrifugation at 6,000 x g for 20 minutes at 4°C, flash frozen, and stored at -80°C.

NSP12/7/8 co-expression:
BL21 *E. coli* were transformed with pG-Tf2 with 20 ng/μl chloramphenicol for selection and made electrocompetent with water and glycerol washes (Au - Gonzales et al., 2013). The resulting cells are designated here as *E. coli* BL21/pG-Tf2. These cells were transformed with pQE-(NSP12)-pcI$^{ts,ind+}$-(NSP7-NSP8), plated on kanamycin/chloramphenicol plates at 30 μg/ml and 20 μg/ml, respectively, and incubated at 30°C. Colonies were inoculated into an 8 ml overnight starter culture which was later inoculated into 1 liter of Terrific Broth + kanamycin + chloramphenicol in a 2.8 liter baffle bottomed flask. The cells were incubated at 30°C with shaking at 250 rpm. When the OD_{600} reached 2, tetracycline was added to 10 ng/ml and the cells were incubated at 16°C with shaking for 20 minutes. IPTG and nalidixic acid were added to 0.5 mM and 50 μg/ml, respectively, and expression was carried out at 16°C with shaking for 16 hours. Cells were harvested and stored as above. Co-expression of histidine-tagged NSP12 and NSP7L8 was performed as for the NSP12/7/8 complex, but with *E. coli* BL21/pG-Tf2 harboring pQE-(NSP12-TEV-8xHis)-pcI$^{ts,ind+}$-(NSP7L8). A nalidixic acid (Sigma Aldrich) stock solution at 50 mg/ml was prepared fresh daily in 0.3 M NaOH.

Protein purification:

Unless otherwise noted, all steps were performed at 4°C. All columns/chromatography resins were purchased from GE healthcare and all dialysis tubing was from Spectra-Por.

NSP8 purification:
Cells were thawed and resuspended in Lysis Buffer (50 mM Tris-HCl pH 8, 2.5 mM EDTA, 150 mM NaCl, 1 mM DTT) at 5 ml buffer per gram cells. Phenylmethylsulfonyl fluoride (PMSF) and lysozyme were added to 10 mM and 0.3 mg/ml, respectively. The lysate was stirred at room temperature for 15 minutes, then sonicated on ice for 20 minutes. Sodium deoxycholate (Sigma) was added to 0.1%, NaCl was added to 0.5 M, and the lysate was clarified by centrifugation at 75,000 x g for 45 minutes. The supernatant was filtered using a 0.2 μm syringe filter (Whatman) and diluted with Dilution Buffer (50 mM Tris-HCl pH 7.5, 0.1 mM EDTA, 10% glycerol, 1 mM DTT) until the conductivity of the sample was equal to the conductivity of Buffer A (50 mM Tris-HCl pH 7.5, 0.1 mM EDTA, 50 mM NaCl, 10% glycerol, 1 mM DTT). The sample was then loaded on 2 x 5 ml Q Sepharose-FF columns with the outlet connected to 2 x 5 ml SP Sepharose-FF columns. The columns were washed with Buffer A and the Q columns were then removed. Proteins bound to the SP columns were eluted with a 200 ml gradient from 10 – 80% Buffer B (Buffer A with 1 M NaCl). Fractions containing NSP8 were pooled, diluted with Dilution Buffer to the conductivity of Buffer A, then loaded onto a 5 ml HiTrap Blue-FF column. The column was washed with 10% Buffer B, then bound proteins were eluted with a 50 ml gradient from 10 – 80% Buffer B. Fractions containing NSP8 were pooled and concentrated with Amicon Ultra centrifugal concentrators with a 10 kDa molecular weight cut-off (MWCO). The concentrated sample was injected onto a Superdex 200 10/300 GL column, equilibrated in 10% Buffer B, and eluted with the same buffer. Fractions containing NSP8 were pooled, concentrated by ultrafiltration as above, then dialyzed into Storage Buffer (50 mM Tris-HCl pH 7.5, 0.1 mM EDTA, 20 mM KCl, 1 mM DTT, 50% glycerol) with 1 kDa MWCO dialysis tubing. Protein concentration was determined by absorbance at 280 nm using the extinction coefficient 19,940 M^{-1} cm^{-1} calculated based on the amino acid sequence of NSP8. Aliquots were flash-frozen in liquid nitrogen and stored at -80°C.

NSP12 8xHis/NSP7L8 complex purification:
Cells were thawed and re-suspended in Lysis Buffer (50 mM Tris-HCl pH 8.0, 500 mM NaCl, 30 mM imidazole, 10% glycerol, 2.5 mM DTT) at 5 ml per gram of cells. One EDTA-free protease inhibitor tablet (Roche) was added with 10 mM PMSF. Sodium deoxycholate was added to 0.1 % and lysozyme was added to 0.3 mg/ml. The lysate was stirred at room temperature for 15 minutes, sonicated on ice for 20 minutes, then clarified by centrifugation at 75,000 x g for 1 hour at 4°C. The sample was filtered through a 0.2 μm syringe filter, then loaded onto a 3 ml HisTrap-FF column, equilibrated in Buffer A (50 mM Tris-HCl pH 7.5, 500 mM NaCl, 30 mM imidazole, 10% glycerol, 2.5 mM DTT). The column was washed with Buffer A until the UV reached baseline, then eluted with a 30 ml linear gradient from 0 – 100 % Buffer B (Buffer A with 500 mM imidazole). Fractions containing the NSP12-His/NSP7L8 complex were pooled, concentrated with Amicon Ultracel 15 centrifugal concentrators (30 kDa MWCO), then dialyzed into Storage Buffer (50 mM Tris-HCl pH 7.5, 50 mM NaCl, 0.25 mM EDTA, 2.5 mM DTT, 50% glycerol) at 4°C with 50 kDa MWCO dialysis tubing. Protein concentration was determined by absorbance at 280 nm using the extinction coefficient 162,850 M^{-1} cm^{-1}, calculated based on the amino acid sequence of each polypeptide assuming a stoichiometry of one NSP12-His to one NSP7L8. The protein was divided into aliquots, flash frozen in liquid nitrogen and stored at -80°C.

NSP12/7/8 complex purification:
Cells were thawed and re-suspended in Lysis Buffer (50 mM Tris-HCl pH 8, 2 mM EDTA, 250 mM NaCl, 10% glycerol, 5 mM DTT) at 5 ml/gram of cells. One protease inhibitor cocktail tablet (Roche) was added along with PMSF, sodium deoxycholate, and lysozyme to 10 mM, 0.1%, and 0.1 mg/ml respectively. The lysate was stirred at room temperature for 15 minutes then sonicated and clarified by centrifugation as above for the histidine-tagged complex. The sample was filtered through a 0.2 μm syringe filter then diluted with Dilution Buffer (50 mM Tris-HCl pH 7.5, 0.1 mM EDTA, 10% glycerol, 5 mM DTT) until the conductivity of the sample was the same as Buffer A (50 mM Tris-HCl pH 7.5, 0.1 mM EDTA, 50 mM NaCl, 10% glycerol, 2 mM DTT).The sample was loaded onto a 30 ml Q-Sepharose-FF column and unbound proteins were removed by washing with Buffer A. Bound proteins were eluted with a 300 ml gradient of 0 – 100% Buffer B (Buffer A with 1 M NaCl). Fractions containing the NSP12/7/8 complex were pooled, diluted with Dilution Buffer, and passed through a 5 ml HiTrap Blue-FF column. The flow-through was loaded onto a 5 ml Heparin Sepharose 6-FF column and unbound proteins were removed by washing with Buffer A. Bound proteins were eluted with a 60 ml gradient of 0 – 100% Buffer B. Fractions containing the NSP12/7/8 complex were pooled and diluted with Dilution Buffer. The sample was flowed through a 5 ml HiTrap SP-FF column equilibrated in Buffer A, and the flow-through was concentrated with Amicon Ultra centrifugal concentrators (30 kDa MWCO). The concentrated sample was injected onto a Superdex 200 10/300 GL column, equilibrated in Buffer A, and proteins were separated in the same buffer. Fractions containing the NSP12/7/8 complex were pooled and dialyzed into Storage Buffer (50 mM Tris-HCl pH 7.5, 0.1 mM EDTA, 50 mM NaCl, 50% glycerol, 1 mM DTT) with 1 kDa MWCO dialysis tubing. Protein concentration was determined by absorbance at 280 nm using the extinction coefficient 181,300 M^{-1} cm^{-1}, calculated from the amino acid sequence assuming one NSP12, one NSP7, and two NSP8 polypeptides per complex (Hillen et al., 2020; Yin et al., 2020). Aliquots were flash frozen in liquid nitrogen and stored at -80°C. Approximately 7 mg of NSP12/7/8 complex was obtained after purification from a one liter expression culture.

Preparation of RNA substrates:

RNA oligonucleotides shown in Figure 1A were synthesized by Integrated DNA Technologies with RNase-free HPLC purification. Oligos were resuspended in Annealing Buffer (10 mM Tris-HCl pH 7, 50 mM NaCl, 0.1 mM EDTA) and the concentration was determined by absorbance at 260 nm using the extinction coefficients 222,360 M^{-1} cm^{-1} and 403,100 M^{-1} cm^{-1} for the FAM-20 nt primer and 40 nt template, respectively. The two oligos were annealed by mixing at a 1:1 molar ratio, heating to 75°C, and cooling slowly to room temperature over approximately 2 hours. Oligonucleotides were stored at -20°C.

Kinetic methods:

Nucleotides were purchased from New England Biolabs. Heparin was purchased from Sigma Aldrich. Chemical structures for figures were prepared with the software ChemDraw and figures were prepared in Inkscape. Remdesivir triphosphate (GS-443902) was provided by Gilead Sciences. Concentration of RTP was determined by absorbance

at 245 nm using the extinction coefficient 24,100 M^{-1} cm^{-1}. The extinction coefficient was measured based on an accurate concentration determined by NMR with an internal formamide standard. Experiments were performed at 37 °C in Reaction Buffer (40 mM Tris-HCl pH 7, 50 mM NaCl, 5 mM $MgCl_2$, 1 mM DTT). Quench flow experiments were performed using a KinTek RQF-3 with reaction buffer in the drive syringes and unless otherwise noted, 0.6 M EDTA in the quench syringe for a final concentration of 0.2 M after quenching.

QUANTIFICATION AND STATISTICAL ANALYSIS

Quantification of reaction products

Kinetics time points were analyzed by capillary electrophoresis on an ABI 3130xl Genetic Analyzer with a 36 cm array and nanoPOP-6 polymer (Molecular Cloning Laboratories) at 65°C. Samples were prepared for analysis by mixing 1 μl of sample with 10 μl of HiDi formamide (ThermoFisher) containing a 28 nt Cy3-labeled DNA oligo internal standard for sizing. Samples were injected for 6 – 12 seconds, depending on the experiment, at 3.6 kV. Peaks area was determined with GeneMapper software and sizing and quantification was performed with a program written in-house.

Analysis of kinetic data

Data fitting/analysis was performed using KinTek Explorer simulation and data fitting software (Johnson, 2009; Johnson et al., 2009b) v10 (www.kintekexplorer.com). This software was also used in preparing figures for kinetic data. Conventional data fitting was performed in the software using built in functions. The equation for a single exponential is $y = A_0 + A_1(1 - \exp(-b_1 t))$, where A_0 is the y-value at time zero, A_1 is the amplitude, b_1 is the decay rate and t is time. The equation for a hyperbola is $y = A_0 + A_1[S]/(K_d+[S])$, where A_0 is the y value at time zero, A_1 is the amplitude and [S] is the x-axis variable. After global data fitting, confidence contour analysis was performed for each data set using the FitSpace function in KinTek Explorer (Johnson et al., 2009a). Parameter boundaries are reported in Table 1, using a χ^2 cut-off in the FitSpace calculation recommended by the software as a limit based on the number of parameters and number of data points used in the fitting of each data set (Johnson, 2019).

From intrinsic rate constants derived in data fitting, we calculated k_{cat}, K_m and k_{cat}/K_m. For example, for the first UTP, we use the following, with $k_1 = 100\ \mu M^{-1}s^{-1}$ (for all fits).

$$k_{cat} = k_2$$
$$K_m = (k_{-1} + k_2)/k_1$$
$$k_{cat}/K_m = k_1k_2/(k_{-1}+k_2)$$

Estimating active enzyme concentration. Estimates of enzyme concentration based on absorbance or dye binding assays can have systematic errors and they do not establish the fraction of enzyme that is active. For these reasons, it is always important to estimate the concentration active sites relative the nominal enzyme concentration obtained from measurements of protein concentration. Because of COVID-19, we have been unable to obtain sufficient RNA templates to perform experiments with duplex RNA in excess of enzyme to provide a better estimate the concentration of enzyme active sites. Nonetheless, our existing data cannot be interpreted without a relatively high fraction of enzyme concentration. Therefore, to extract estimates of the minimal enzyme concentration necessary to account for the kinetic data, we added a step to the pathway to allow for a very slow equilibration between active (E) and (X) dead enzyme as shown in the pathway given in Figure 3, reproduced here.

$$X \underset{K_{active}}{\overset{slow}{\rightleftharpoons}} E + R_{20} \underset{k_{off}}{\overset{k_{on}}{\rightleftharpoons}} ER_{20} \underset{k_{-1}}{\overset{k_1\,[UTP]}{\rightleftharpoons}} ER_{20}.UTP \xrightarrow{k_2} ER_{21} \underset{k_{-3}}{\overset{k_3\,[UTP]}{\rightleftharpoons}} ER_{21}.UTP \xrightarrow{k_4} ER_{22}$$

We do not imply that the reaction between E and X is reversible, only that we can model it as such to take advantage of the power of global data fitting and confidence contour analysis to estimate the fraction of enzyme in the active state. We set the rate constants for E – X equilibration to be on the order of 1e-05 s^{-1} and allowed the reaction to equilibrate (computationally) on for 500,000 s (~6 days). The equilibrated enzyme was then mixed with RNA for 250 s, which is long enough to allow RNA binding only to the E form without perturbing the E – X equilibration. In a third

mixing step in the software, we then added nucleotide to follow the fast reaction (< 1 s). In fitting the data, we locked the rate constant for E $\rightarrow$ X at 1e-05 s^{-1} and then allowed the reverse rate constant to float in globally fitting the all of the data shown in Figure 3. Confidence contour analysis showed that each of the rate constants governing binding and incorporation of the first and second UTP were well constrained by the data. Figure 3F shows the results from systematically varying the rate constant for the X $\rightarrow$ E reaction in order to compute a hypothetical equilibrium constant, K_{active} = [E]/[X] so we than calculate the fraction of active enzyme as [E] = $K_{active}/(1+K_{active})$, which we plot in the confidence contour shown in Figure 3F. These data show that there is a clear threshold at 60% active enzyme (relative to the nominal concentration). Below this value, the data fit gets significantly worse as judged by an increase in χ^2 (decrease in χ^2_{min}/χ^2). With increases in the fraction of enzyme that is active past 0.6, the data fit gradually gets better, but there are no significant changes in the value of χ^2 or in the values of parameters derived in fitting data. It should also be noted that the calculated second order rate constant for RNA binding shows an inverse correlation with the fraction of active enzyme over the narrow range from 60% to 100% active enzyme. This is consistent with an understanding that in experiments with enzyme in excess, the pseudo-first order rate constant for RNA binding will be given by the product of k_{on}[E]. This also leads to a modest change in the rate constant for RNA binding.

We then locked the fraction of active enzyme at 80% and then fit the titration (Figure 3D) and RNA dissociation (Figure 3E) globally to define the rate constants for RNA binding and dissociation, with confidence contours shown in Figure 3G. Similarly, we fit the UTP binding and incorporation results (Figures 3A, B, and C) with 80% active enzyme to get the confidence contours shown in Figure 3H. Data fitting for all of the figures in the paper, including Figure 3 was obtained using a nominal active enzyme concentration equal to 80% of the value estimated by absorbance measurements.

Supplemental References

Aslanidis, C., and de Jong, P.J. (1990). Ligation-independent cloning of PCR products (LIC-PCR). Nucleic Acids Research *18*, 6069-6074.

Au - Gonzales, M.F., Au - Brooks, T., Au - Pukatzki, S.U., and Au - Provenzano, D. (2013). Rapid Protocol for Preparation of Electrocompetent Escherichia coli and Vibrio cholerae. JoVE, e50684.

KEY RESOURCES TABLE

REAGENT or RESOURCE	SOURCE	IDENTIFIER
Bacterial and Virus Strains		
BL21 *E. coli*	New England Biolabs	Cat#C2530H
Chemicals, Peptides, and Recombinant Proteins		
Isopropyl-beta-D-thiogalactoside (IPTG)	Gold Biotechnology	Cat# I2481C
Nalidixic Acid	Sigma Aldrich	Cat# N8878
Tetracycline	Fisher Scientific	Cat# BP912100
SARS-CoV-2 NSP8	This paper	N/A
SARS-CoV-2 NSP12/7/8 complex	This paper	N/A
Oligonucleotides		
RNA Primer: [6-FAM]-[6-FAM]-GUCAUUCUCCUAAGAAGCUA	Integrated DNA Technologies	N/A
RNA Template: CUAUCCCCAUGUGAUUUUAAUAGCUUCUUAGGAGAAUGAC	Integrated DNA Technologies	N/A
Cy3 Internal Standard DNA Oligo: [Cy3]-CCGTGAGTTGGTTGGACGGCTGCGAGGC	Integrated DNA Technologies	N/A
Recombinant DNA		
pcI$^{ts,ind+}$-(NSP8)	This paper	Deposited to Addgene --160656
pQE-(NSP12)-pcI$^{ts,ind+}$-(NSP7-NSP8)	This paper	Deposited to Addgene -- 160540
Software and Algorithms		
KinTek Explorer v10	KinTek Corp	https://kintekexplorer.com
Chemdraw Professional v16	Perkin Elmer	https://www.perkinelmer.com/product/chemdraw-professional-chemdrawpro
Inkscape v0.92	Inkscape	https://inkscape.org/
Gene Mapper v5	ThermoFisher Scientific	https://www.thermofisher.com/order/catalog/product/4370784#/4370784
SnapGene Viewer v5.1.5	GSL Biotech	https://www.snapgene.com/
